# An Economic Analysis of the Shading Effects of Transmission Lines on Photovoltaic Power Plant Investment Decisions: A Case Study

**DOI:** 10.3390/s21154973

**Published:** 2021-07-21

**Authors:** Henrik Zsiborács, Nóra Hegedűsné Baranyai, András Vincze, Gábor Pintér

**Affiliations:** 1 Renewable Energy Research Group, Soós Ernő Research and Development Center, University Center for Circular Economy, University of Pannonia Nagykanizsa, 8800 Nagykanizsa, Hungary; zsiboracs.henrik@uni-pen.hu (H.Z.); vincze.andras@uni-pen.hu (A.V.); pinter.gabor@uni-pen.hu (G.P.); 2 Smart-Inno Expert Kft, 8360 Keszthely, Hungary

**Keywords:** solar energy, photovoltaic field inspection, hot spot phenomenon, shading, transmission lines

## Abstract

In today’s photovoltaic (PV) power plants, traditional crystalline PV modules are the prevalent technology, which is highly susceptible to partial shading due to the risk of irreversible damage. Therefore, it is advisable to explore potential construction sites for objects that might cause shading, including high-voltage transmission towers, whose shading effects can be significant due to their height. By means of innovative simulation, using a model, validated with actual data, this study endeavored to deliver novel information related to the problems of shading by high-voltage transmission lines. In the context of Hungary, it examined the risk factors, technical and economic aspects, and possible solutions important for PV projects. It provides new insight, much needed also at the international level, considering the fact that the extent of the shadows cast by conductors on the surface at low Sun elevations is not known at present and neither are the shading characteristics of conductors between two transmission towers, depending on their height, in winter, when the Sun is low. An added practical benefit of the study is that its technical and economic approaches and the software solutions are all based on the practice of PV system design and construction. Related to the investigated issues, this can facilitate the formulation of the technical and economic aspects of suitable PV power plant building strategies in Hungary.

## 1. Introduction

### 1.1. The Spread of PV Systems around the World and Various Aspects of Their Designs

PV systems play an increasingly important role in the world’s energy supply; at the end of 2018, they were responsible for 2.4% of the global electricity generation, while, at the end of 2019, the figure was 2.8%. By the end of 2019, the total global PV capacity approached 630 GW, and, in the same year, the cumulative PV capacity of Bulgaria, Denmark, and Hungary exceeded 1 GW [1]. According to certain scenarios, it is expected that, by 2022, the capacity of global installed PV systems will be between 824 and 1290 GW, and it will be between 1043 and 1610 GW by 2023 [2]. In the country studied herein, Hungary, a decrease in the number of fossil fuel power plants and an increase in the significance of nuclear energy are expected by the Hungarian Transmission System Operator (MAVIR), while, among the variable sources of renewable energy, it is PV systems whose role is projected to become more and more decisive in the next two decades. MAVIR’s data show that the total installed PV capacity at the end of December 2019 was 1.3 GW, which will surpass 6 GW in 2030 and may reach 12 GW in 2040 [3]. These ambitious targets project that PV power plant investments will come more and more to the fore.

The correct orientation of PV modules and the selection of the optimal tilt angles during the design of a PV power plant are some of the most important tasks for ensuring the efficient operation of the system [4]. If the orientation and the tilt angle are not chosen well, the PV power plant does not produce the amount of electricity it could, compared to a more appropriate (more rational) setting; consequently, the economic indicators of the investment may also deteriorate significantly, e.g., the payback time may be considerably longer. Additionally, being aware of the climatic and other individual conditions of the location (e.g., topography) is indispensable for the maximization of the amount of energy that can be generated annually [5,6]. This also means that the ideal settings (e.g., the distances between strings) are different from place to place. In Europe, the ideal tilt angle for south-facing PV systems ranges between 20° and 50° [7].

In new investments, among the conventional PV technologies, it is the monocrystalline one that has enjoyed priority since 2016 [8]. This market trend is becoming more and more dominant [8] since, on the one hand, the module cost of the monocrystalline technology expressed in EUR/Wp is practically the same as that of the polycrystalline one [9], and, on the other hand, sites selected for plant investments can be used more efficiently [8] when using monocrystalline PV modules due to their higher efficiency [10].

### 1.2. The Characteristics of Conventional Crytalline and Maxim Integrated (MX) PV Technologies Related to Shading

The disadvantage of conventional crystalline PV technologies is that the module cells are sensitive to nonideal effects [11] that cause differences in performance among the PV cells [12]. Such negative effects can be caused by local environmental conditions [13,14], mismatch losses [15], contamination by dust, dirt, and bird/insect excreta, and shading [13]. It is important to note that, when the conditions under which a PV array is working is homogeneous, there can only be one maximum power point (MPP), and that allows easy MPP tracking. However, in the case of varying conditions of operation, for example, caused by partial shading, the same PV array may also have different MPPs, resulting in the fact that the values of the global MPP can vary to a high degree on the voltage range [16]. From the perspective of investment, partial shading is a significant risk factor because of the potential damaging of the PV modules [17]. In traditional crystalline PV technologies, partial shading results in PV cells operating as electric load, and the electric power is transformed into heat. In such a case, PV cells can be imagined as current sources; thus, in the event of differences between the currents of the cells connected in a series, strongly localized dissipation occurs. This, in turn, causes a rise in the temperatures in the PV cells, causing so-called hot spots (hot cells) or hot strings [11], which may result in permanent damages in the PV module [18]. The manufacturers of PV modules know this phenomenon well, and that is why they use bypass diodes, which are placed in the junction boxes to eliminate the strings in which there are PV modules with reduced performance from the joint electricity generation, thus decreasing the dissipation in the shaded cells and the risk of sustaining damage. Conventional crystalline PV modules of greater power typically contain three series of cells and three bypass diodes. These diodes have their operating points, which have to be reached; otherwise, no electricity can be diverted, i.e., the cells will consume a part of the generated energy, creating [19] a hot spot phenomenon [20]. The electric characteristics related to the partial shading of PV modules have been treated in detail in several articles [21,22,23,24,25,26,27,28], and it has been established that this phenomenon [29] leads to a higher rate of PV module failures and the accelerated aging of cells [11]. It is important, however, to mention that, at present, there is already a technological solution [20] that can efficiently handle the hot spot phenomenon [30] and the problems of loss by shading [31] in the case of crystalline PV modules [32]. The real breakthrough in the efforts to significantly reduce losses caused by shading was brought about by the appearance of Maxim Integrated’s optimizer integrated circuits (IC) on the market. In PV modules equipped with these, performance optimization is done already at the level of the series of cells, since the ICs are installed in the place of the diodes [33]. Thus, in PV modules with integrated cell-string optimizers, the three series of cells operate as separate parts, whereby each string is capable of delivering maximum performance. This solution can greatly improve the long-term reliability of PV modules by preventing the hot spot effect and minimalizing the effects of the aging process of cells [12]. In 2021, PV modules marked MX, manufactured by Suntech Power Co., Ltd. (Wuxi, China) [34] and JinkoSolar Holding Co., Ltd. (Shanghai, China), feature cell-string optimizers [35,36]. These PV modules are globally available to investors in the market [34,35,36].

### 1.3. Practical Features of Hungarian PV Power Plant Investments and Their Relationships with the Study Area

In Hungary, the typical capacity of new PV power plants was between 0.5 and 42 MW in 2020. In 2021, however, investors already seem to favor greater PV investments (at least 15 MW). For these, they mostly choose conventional crystalline PV technologies [10] because of their more favorable prices [9]. A consequence of the trend of creating greater PV capacities is that new power plants require ever-growing land areas. It also follows naturally that investors tend to demand that the selected sites be surveyed from every aspect in order to identify all risk factors (e.g., shading by trees or transmission lines) [10,37,38]. From the perspective of shading, high-voltage transmission towers and their conductors can greatly influence the profitability characteristics of PV power plants all over the world, as these objects have extremely great and significant shading effects. At present, the extent of the shadows cast by conductors on the surface at low Sun elevations is not known and neither are the shading characteristics of conductors between two transmission towers, depending on their height, in winter, when the Sun is low [10,37,38]. Research results related to this issue are currently not yet available for Hungary and are also rather scarce at the global level. This is the reason why this study endeavors to explore, in connection with the problems of shading by high-voltage transmission lines, the risk factors important for PV power plant investments, the technical and economic aspects, and the possible solutions, using a Hungarian example. The novel practical benefit of the manuscript lies in the fact that the technical and economic approaches and the software solutions are all based on the practical experience of the design and construction of PV systems, which can be of help in the formulation of the technical and economic aspects of suitable PV power plant building strategies in Hungary, related to the investigated issues.

## 2. Materials and Methods

### 2.1. High-Voltage Transmission Lines in Hungary and the Various Aspects of Shading

**Figure 1:**
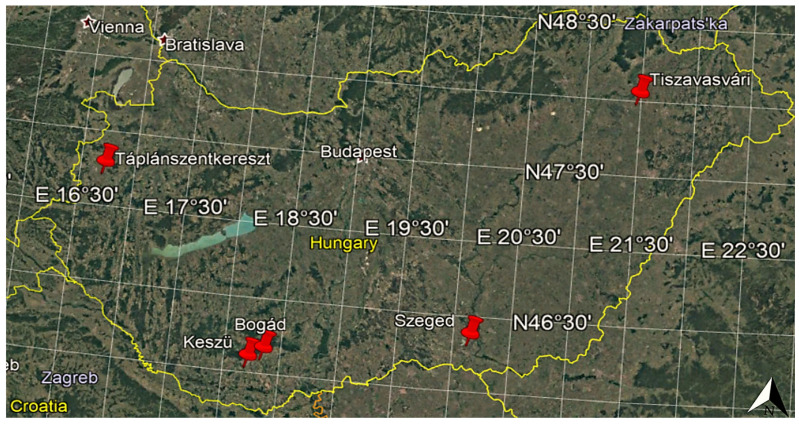
The locations selected for the examination of the shading effects of high-voltage transmission towers and their conductors (sites indicated by the red markers), based on [41].

According to official data, the route length of the Hungarian high-voltage network is 3724 km [39]. The total network length of all the high-voltage transmission lines, the characteristic tower types, and the exact conductor types are described in great detail in the source provided by MAVIR [40], and a number of technical parameters and characteristics can be established on their basis. This manuscript takes the network length of the high-voltage transmission lines as a basis for determining how common the various high-voltage transmission tower types are. According to the document, the numbers and typical heights of the high-voltage transmission tower types were established, and, through the analysis of the network lengths of the transmission lines, the tower types used for various line lengths could also be identified. It could also be established [40] that, in the case of a given network length, several tower types of different technical parameters were installed in certain cases in Hungary (e.g., Albertirsa-Szolnok Ovit, 3 pcs over 45.6 km; Győr Ovit-Szombathely, 2 pcs over 112.7 km). In these cases, however, no further information was available about the line lengths of the different tower types. This is the reason why, in the cases when several tower types belonged to a given network length, the distance was proportionally divided by the number of the towers of the given tower types. With the help of the applied method, the nature of the distribution of the tower types used in Hungary could be determined with great accuracy on the basis of the total network length of the high-voltage transmission lines. This information was important because the examinations herein took the most common tower type into consideration in connection with PV power plant investments. The shading characteristics of the high-voltage transmission towers and their conductors were determined using the Google Earth Pro software [41], which made it possible to view detailed, high-resolution satellite images of the Earth’s surface. In the course of the investigations, five Hungarian locations (Bogád: 46.092998, 18.321933; Táplánszentkereszt: 47.212153, 16.701414; Tiszavasvári: 47.976401, 21.456783; Szeged: 46.306404, 20.121326; Keszü: 46.031793, 18.191574), whose high-resolution satellite images demonstrated the shading effects in a clearly visible way, were explored in detail, and, with their help, general conclusions could be drawn for Hungary. In these locations, the distances between the high-voltage transmission towers ranged from 250 to 400 m. Thus, the average distance of 325 m was used for modeling purposes. The examined satellite images, which could be found for all the sites in the Google Earth Pro software, were taken between 2012 and 2020. The main criterion of the selection of the sites was that they had to be located in different parts of Hungary (Figure 1). Phenomena similar to the shading effects of Hungarian high-voltage transmission towers and their conductors can be observed both in the northern (Sweden, Botkyrka S, 59.114852, 17.834234) and in the southern (Chile, Buin, −33.687995, −70.696215) hemispheres. As shown in Section 3.2, it can be stated that high-voltage transmission towers and their conductors cause similar shading issues in both hemispheres.

### 2.2. The Hungarian Regulatory Environment Related to PV Power Plants—An Overview

The 2000s witnessed the dynamic spread of the use of the renewable sources of energy. As a consequence, individual countries created their own systems for promoting green energy [8], which are, however, far from uniform and show great differences. These support systems also change dynamically, following the development of renewable technologies [42].

The Hungarian system of supporting green energy from renewable energy sources (KÁT) [43] is a tool for promoting the generation of electricity from renewable energy sources and waste in Hungary, according to which the electric power can be sold [44] at the price determined by law, which is higher than the current market price. The key feature of the KÁT system is that, by determining the eligible amount of electricity and the period of eligibility, producers can be guaranteed to receive support only during the payback period. It is important to mention that the period of eligibility is reduced proportionally if the power plant receives any other support. No eligibility can be granted for applications for KÁT support submitted later than 1 January 2017 [45].

The Renewable Energy Support Scheme (METÁR) [46] was launched in Hungary on 1 January 2017. Under this regulation, only those renewable energy production investments may be granted support [47] whose realization has not been started at the time of applying for it. Another feature of this scheme is that power stations burning waste and/or mixed fuel can only receive support in proportion to their use of renewable energy sources. Household-sized photovoltaic power plant systems up to 50 kW of power (fed in to the grid) are an exception, since this legal category is not allowed to receive support in this form at all. A further condition for support under the METÁR system is that applicants have to have the green premium type eligibility granted to them during the application procedure. Those who are granted the support sell the electric energy themselves via MAVIR, and they also have to bear the costs of any deviation in their electricity production from the 15 min schedule [48]. The first call for applications was announced by the Hungarian Energy and Public Utility Regulatory Authority (MEKH) in the autumn of 2019 [49]. The lion’s share of the applications received was related to PV technology [50]. The winning applicants of the METÁR scheme receive the selling price proposed by them 15 years long, which is indexed to the rate of inflation decreased by one percentage point annually, and they have to sign a contract with MAVIR. According to market expectations, indexing below the rate of inflation was applied, since the operating costs are expected to increase below the inflation rate thanks to technological developments. For example, the replacement of an inverter due in 8 years is likely to involve lower expenses in real value than today. In the category of larger plants (units of at least 1 MW), the applicants’ proposed prices ranged between 0.056 and 0.063 EUR/kWh in 2019 [49]. For the economic calculations herein, the average proposed price of the successful applications weighted by MEKH was used, which was 0.059 EUR/kWh. The economic calculations of this article were based on the METÁR regulation [46].

### 2.3. The Characteristics of the Simulation Procedure Used in the Model, Validation, and the Description of the Aspects of Modeling

Today, in the realization of PV power plant investments, the application of the adequate PV software is indispensable for the simplification of the designing process. There are numerous PV system designing applications, which are capable of performing different modeling and simulation tasks [51]; thus, it is crucial to choose the software that suits the given investment best. In the course of designing a PV power plant, it is of utmost importance to explore the specific conditions of the potential sites thoroughly (e.g., topography, trees, shading objects), since this is how the useful area can be increased (e.g., by landscaping) and the risk factors decreased. Moreover, this information also influences the economic indicators of the investment. During the planning of PV power plants, it is a common difficulty that numerous applications prepare their models projected onto a plane, i.e., they do not take the relief of the location into consideration. This is important because a hillier area changes the shading effects affecting the PV strings compared to a level surface. Understanding the relief features of a site is extremely important for the optimal row spacing of strings because it is a requirement in PV power plant design in Hungary that the annual loss in energy yield resulting from the mutual shading of the PV modules must not exceed 3% [10,52]. This is why the highest position of the Sun at the winter solstice is taken into consideration [53] when determining the row distances between the strings [10]. Furthermore, for establishing the shading effects of the objects that might be problematic for the site, the time from 9:00 a.m. to 2:00 p.m. (local time) at the winter solstice is decisive [37].

One of the popular software solutions for designing PV power plants is using SketchUp Pro [54] with its plug-in Skelion [55]. The simulation procedures used for modeling in this paper were created with these programs. Sketch Up Pro is a 3D modeler, which is widely used in the fields of architecture, mechanical engineering, the film industry, and video game design. The software allows the import of high-quality, high-resolution maps of any geographical location from Google Earth. It can display the precise layout or relief of any examined site, the images can be edited, and models can be made on them. Using SketchUp Pro allows users to determine the shading effects of any object at any geographic location accurately at any time to the minute. Moreover, the program can calculate the area of the examined site or the surface area or volume of any 3D object. It can be of great help with the design of a PV power plant by facilitating the thorough exploration of the potential construction site, since, by using it, one can achieve the following:

- Create relief-specific models for the given location;
- Accurately see the objects posing shading problems with the help of satellite images imported from Google Earth, and reconstruct them as 3D objects with great precision;
- Change the shading effects of the problematic objects created as 3D objects as a function of time in an interactive way, which helps identify areas not suitable/problematic for the placement of PV modules;
- Determine the useful area of the potential construction site.

Skelion is a plug-in of SketchUp Pro, which allows the creation of a 3D PV system. It is capable of designing PV systems for any object, i.e., level or uneven areas. Its advantage is that it can provide high-accuracy estimates for the size of the PV system that can be placed at the potential site, the expected energy production in the first year (kWh/kWp), and the number of the PV modules, and it can also be complemented with any further technical characteristic of the PV modules. Creating models, the program also takes the databases of the Photovoltaic Geographical Information System [56] and the National Renewable Energy Laboratory [57] into account. The data from these are based on weather data series of 10 years in the case of the former and several decades in that of the latter. During modeling, it is also possible to set the desired orientation and tilt angle, the arrangement of the rows, and vertical or horizontal module mounting. When placing PV strings on a given relief, the model considers the shading effects between the rows by string, according to the specific geographical and climatic conditions based on the highest position of the Sun at the winter solstice. The model provides help with the optimal determination of the inter-row spacing at the site. One of the main advantages of the program is that it can make calculations for each PV module separately, considering the hourly, annual shading effect of any object, as well as assess, in the form of a report, the energy loss resulting from the annual shading, at both module and system levels. The report also contains data in a monthly breakdown; hence, there is also a possibility to formulate more precise conclusions. It is also possible to visualize the annual energy loss resulting from shading in 3D at module level. Thus, it is also easy to identify areas that are most prone to shading.

In the course of the investigations herein, the annual energy loss of the PV modules due to shading was determined with the help of the Skelion program. During the modeling, the unshaded and shaded zones of the total area were separated, making it was possible to distinguish the energy generation potentials of both sub-areas by month and to assess the amount of annual energy loss. The term “shaded zone” refers to areas where problematic objects cause shading of different degrees during the year, while problems of this kind do not occur in unshaded areas. This research presupposed that, in the case of the Maxim Integrated PV modules exposed to shading, the annual energy loss due to shading would be 2% less than that in the case of the conventional PV modules [32]. This correction value was also taken into consideration in Skelion. In the course of the modeling, according to the literature and professional experience, the annual performance degradation of unshaded crystalline PV modules was determined as 0.5% [58,59], while, in the case of shaded locations, that of MX PV modules was determined as 0.8% and that of conventional PV modules was determined as 2.5% [20,32,60]. In the case of traditional PV technology mounted in a shaded area (scenarios B 1, C 1) a failure rate/replacement need of 3% every 3 years was taken into consideration [10,38]; thus, the model also took the replacement and selling of defective PV modules into account (Table 1).

In PV power plant constructions, the most commonly used protective distance in the case of fences is 5 m, while, in the case of high-voltage transmission lines, the protective distance from the conductor is 18 m in both directions. It is not necessary to take the shading effects of the 2 m high fence into account. The reason for that is that its shaded zone can only reach the base of the mounting structure at most in the case of PV power plants, as the bottom part of the PV modules is at least at a height of 1 m from the ground [37]. Furthermore, an electrical substation also has to be provided with a safety zone within a 10 m^2^ area measured horizontally from the edge of the fence [10,61]. This paper calculated with a 40 m^2^ area (4 × 10 m^2^) because of the area needed by other devices (e.g., inverter). These values were used herein when creating the models (Table 1).

The validation of the correct functioning of the Skelion and SketchUp Pro programs used for the modeling was carried out with the help of a PV power plant with a nominal power of 0.5 MW located at a site in Kiskölked, Rádóckölked (47.069888, 16.580578; approximately 0.67 ha) in Hungary. It was necessary to confirm that the modeling software was suitable for the credible assessment of the potential sizes of PV power plants at individual sites. Upon validation, the capacity of the PV power plant created by the modeling method during the modeling procedure at the selected location showed agreement with the power of the actual PV power plant. Thus, it was proven that the methodology used was suitable for the reliable assessment of the potential sizes of PV power plants at individual locations. The PV system had a southern orientation (180°) and the tilt angle of the PV modules was 30°. Using the two applications, the site of the PV power plant with the installed PV modules (Canadian Solar, CS6U-330 [62]) could be recreated with high precision, and it proved to be suitable for the modeling of the whole PV power plant.

**Figure 2:**
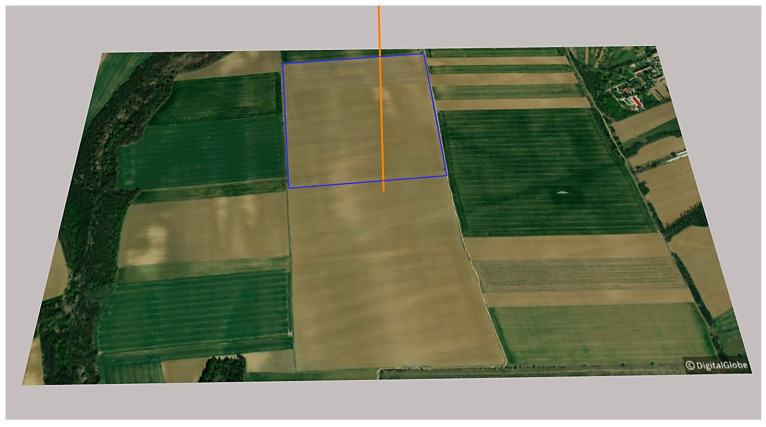
The site selected for modeling: Juta, 46.403736, 17.719469 (area with blue outlines: the examined 22.7 ha area; orange line: north), based on [54].

The locations of PV power plant investments are unique, showing various differences. For this reason, this study examined a randomly selected ideal location occupying 22.7 ha, with identical sides (476 × 476 m), an almost optimal north–south orientation, nearly level relief, and no shading by problematic objects. One of the research objectives was to examine how the high-voltage transmission towers and their conductors, problematic from the perspective of shading, affect the feasibility of larger PV power plants and the profitability features of the investment in the selected 22.7 ha area. For this, first of all, the abovementioned nearly ideal location was chosen (Juta, 46.403736, 17.719469) (Figure 2).

In this research, the possible installation solutions of both conventional and MX PV modules were examined (Table 1). The PV modules studied herein were all of the type using monofacial monocrystalline technology. For the greatest possible accuracy of the results of the examinations related to the subject matter of the study, it was necessary to input the technical parameters belonging to actual, selected types of PV modules (e.g., weight, width, and length) into the modeling software, as different PV modules could produce differing results. In the case of the conventional PV technology, the Jinko Cheetah JKMS315M-60B was selected, while, in the case of the MX solution, the Jinko Cheetah Maxim Integrated JKMS320M-60HB-MX3 was selected. The choice of these particular PV modules was justified by the fact that they are both from the same manufacturer, their sizes and weights are the same, and their electric characteristics are identical to a significant degree. This makes designing the strings for the inverters easier in the case of combining the PV modules, since the mounting does not need altering for different PV module sizes. Furthermore, they are easily available in the market in 2021.

The research investigated three different scenarios (an ideal one and two extreme ones), as also shown in Table 1:

1. Selected site without shading problems affecting potential use for PV power plant (ideal location, scenario A);
2. Selected location with transmission towers and their conductors traversing the site in an east–west direction, cutting the area into two halves down the middle (one of the worst conditions for a location, scenario B);
3. Selected location with transmission towers and their conductors traversing the site in a north–south direction, cutting the area into two halves down the middle (one of the worst conditions for a location, scenario C).

Seven scenarios were enough to present the goals, innovative novelty, and the new practical benefits of the study. In scenario C, the length of the high-voltage transmission line crossing the area in a north–south direction was the same as that of the transmission line traversing the site in an east–west direction in scenario B. In the model, the transmission tower was located in the middle of the area (238 m), and the next one was at a distance of 325 m in an east–west or north–south direction. The modeling and technical aspects of the three scenarios are detailed in Table 1.

In the course of the investigations, the following hypotheses were verified:

- Due to their shading effects, high-voltage transmission towers and their conductors affect the layout characteristics of PV power plants and their performance.
- When the shading effects of high-voltage transmission towers and their conductors are taken into consideration in designing a PV power plant, and when MX PV modules are also installed in the shaded area, a difference can be observed in the specific energy generation and the performance of the PV power plant.
- When the shading effects of high-voltage transmission towers and their conductors are taken into consideration in designing a PV power plant, and when MX PV modules are also installed in the shaded area, a difference can be observed in the payback time of the PV power plant investment.
- Both the performance of PV power plants and the payback time of investments are influenced by the direction (east–west or north–south) in which the high-voltage transmission towers and their conductors traverse the area.

### 2.4. Methods and Details of the Economic and Technical Assessment

Well-grounded decisions concerning PV system investments can only be made if their feasibility is supported by economic calculations (Table 2). In the case of ground-mounted systems, a great dilemma facing investors is posed by the need to take the transmission towers and their conductors into consideration. When building a PV power plant, just like in the case of any other investment in the electricity sector, high-value and long-life installations are created. A common feature of such investments is that they involve significant costs, their returns occur only later in time, and their value is not known with full certainty in advance [63,64].

The economic calculations in this paper were based on the METÁR regulation [45,47,49,50] and applied the successful proposed price (weighted average) approved of by MEKH at the end of 2019. For every year, the proposed price was indexed to the rate of inflation decreased by one percentage point. Following the first year, changes in the inflation were taken into consideration at the value of 5 years (2015–2019) average inflation [65] rate (2.29%). In the course of the modeling, the nominal values of each of the annual expenses were determined using this future rate of inflation (2.29%) on the basis of the real values of the expenses of the given year. Thus, the model presumes that the real values of the expenses incurring each year remain unchanged. The paper examines an investment period of 15 years, since, on the one hand, successful MEKH applicants receive the price proposed by them 15 years long (thus, the yield can be estimated accurately for this period), and, on the other hand, after 15 years, the technical condition of the PV modules and the inverters can still be regarded as good (thus, they can be sold at acceptable prices). This economic approach makes it possible for investors to use new and more efficient technologies every 15 years; at the same time, PV technology in adequate technical conditions also becomes available to less privileged social strata [66]. The interest rate used in the model reflects the status of the 15 year Hungarian government bond of 22 December 2020 [67]. This research presumes the selling of the PV modules, inverters, and mounting structures in the 15th year. In the case of the PV modules [68] and inverters [69], the secondhand prices prevalent in the fourth quarter of 2020 were taken into account, while, in the case of the mounting systems, this was 25% of the selling price of the PV modules [70]. Regarding the selling of the equipment, the price prevailing in the fourth quarter of 2020 was adjusted with the inflation rate occurring during the 15 year period, i.e., the model presupposes that the real value of the market price remains unchanged [66]. The model also considers selling of those PV modules that are replaced every 3 years. For the conversion of the prices from HUF to EUR and vice versa, the reference exchange rate of 22 December 2020 was used [71].

In Hungary, the price of the PV modules constitutes approximately 40% of the costs of the complete realization of a PV power plant, while the other expenses amount to 60% [10,70,72]. For every modeling, the number of PV modules can be determined with the help of the Skelion program; knowing the area, the number of the PV modules, and their prices, the costs of the PV power plant investments can be calculated for each scenario. The Jinko Cheetah JKMS315M-60B module, used in the model, was available in Europe for EUR 104 [35], while the MX Jinko Cheetah Maxim Integrated JKMS320M-60HB-MX3 module costed EUR 129 [36] in the fourth quarter of 2020. In the model (scenarios B1 and C1), extra PV module expenditure was presumed because of module replacements every 3 years due to the traditional PV technology installed in shaded areas. The investment costs of the PV systems included the costs of the extra modules [10,70,72]. The reference price of the operating and maintenance tasks of the PV power plants was estimated at 3000 EUR/MWp on the basis of practical experience [73].

From an economic perspective, the thorough exploration of the unique features of a potential construction site is of extremely great importance. It is important to note here that the purchase of land is an expense that occurs in addition to the installation and complete realization of a PV power plant. Since the value of land can be regarded constant in the long term, it can be sold at the end of the useful investment period at the same real value as it was purchased originally. In the course of the investigations, the expenses associated with the purchase and selling of properties were not taken into consideration [37].

In Hungary, every commercial producer is obligated to submit day-ahead and intraday schedules to MAVIR. From 1 January 2020, the producer is responsible for keeping the schedule in a disciplined way and paying for any balancing energy. Because of the complexity of this, PV power plant owners turn to companies that prepare their schedules, take responsibility for the costs of balancing energy, provide full administration, and conduct inverter monitoring. In the fourth quarter of 2020, such a service costed EUR 110 per 500 kWp PV system. However, this is expected to increase by 10–25% annually until 2026 as a result of changes in the legal regulation. The reason for this is that, in the case of those submitting the schedules, the calculated surcharge is mitigated by a unified surcharge reduction; however, this protective mechanism will terminate in 2026. From 2026, the whole surcharge will be payable by those submitting the schedule [37,74,75]. The model assumed a 10% increase of the costs annually until 2026, based on the suggestion of PANNON Green Power Ltd. [37] (Table 3).

In the case of the economic calculations only the dynamic indicators were determined, since the time value of money was an important aspect in the modeling; the paper did not use static indicators. Among the dynamic indicators, the net present value (NPV), the internal rate of return (IRR), and the discounted payback period (DPP) helped draw important conclusions related to the investment characteristics of the examined systems (A, B1, B2, B3, C1, C2, and C3, Table 1) [64,66]. The economic aspects applied in the model are shown in Table 3, while the context of the calculations is presented in Table 2.

## 3. Results

### 3.1. The Most Common High-Voltage Transmission Tower Types in Hungary

According to the document provided by MAVIR [40], the numbers and typical heights of the high-voltage transmission tower types were established using the methods presented in Section 2.1.

According to official data, the route length of the Hungarian high-voltage network is 3724 km [39], but the network length of all the high-voltage transmission lines is 4869 km. The reason for this discrepancy is that, in certain sections (e.g., Albertirsa-Göd I-II, Szombathely-Hévíz I-II.), a double network length was built. In Hungary, altogether, 39 different types of high-voltage transmission towers can be found, and their heights range between 20.5 and 53.2 m. Their other characteristics are given in Table 4.

As a function of the total network length of the high-voltage transmission lines, the distribution of the 39 tower types in use was determined. According to the results, the three most common types are Fenyő, Ipoly, and Kaposvár. Their main characteristics are outlined below.

- Fenyő: frequency: 22.28%, height: 46 m, outer diameter of conductor: 31.05 mm;
- Ipoly: frequency: 9.56%, height: 35.5 m, outer diameter of conductor: 31.05 mm;
- Kaposvár: frequency: 9.01%, height: 33 m, outer diameter of conductor: 31.05 mm.

The diameter of the conductors is 31.05 mm [80] for all three types. The study based the calculations related to PV power plant investments on the most common tower type, Fenyő, with a height of 46 m and an area requirement of 12.8 m. In the model, the first tower was located in the middle of the area [40].

### 3.2. The Shading Characteristics of Hungarian High-Voltage Transmission Towers and Their Conductors

**Figure 3:**
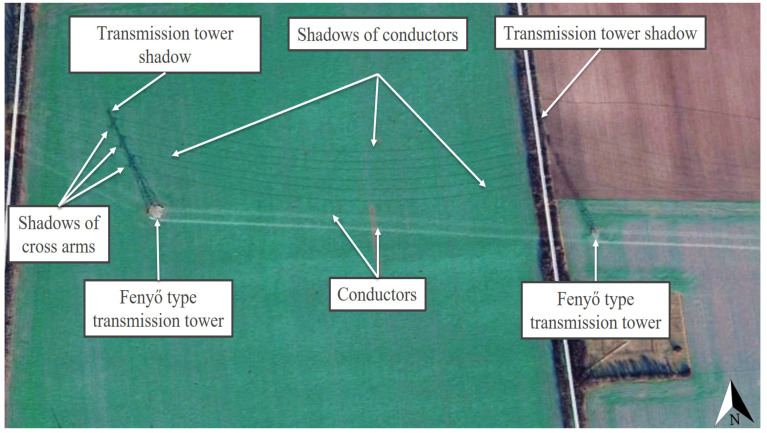
The shading effects of Fenyő-type transmission towers and their conductors on 2 January 2020 at approximately 11:30 a.m. (local time) in Táplánszentkereszt, based on [41].

**Figure 4:**
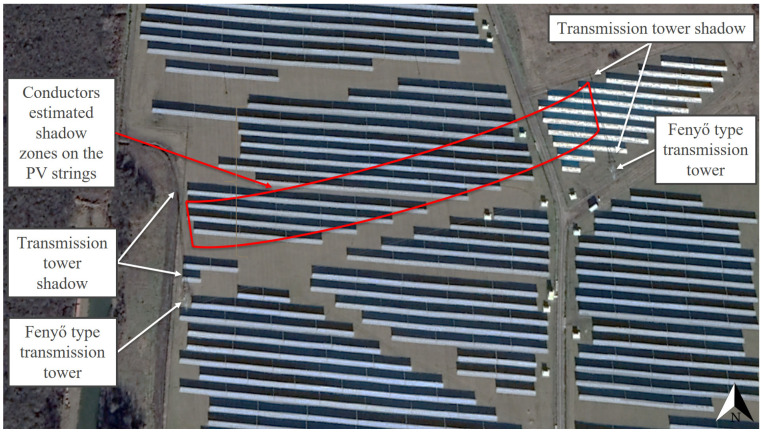
The layout of an 11 MWp PV system with high-voltage transmission towers and their conductors in Keszü (46.031793, 18.191574) on 2 February 2020 at approximately 11:30 a.m. (local time), based on [41].

For the illustration of the shading effects of the high-voltage transmission towers and their conductors and the relevant situation of PV investments, the locations Táplánszentkereszt (47.212153, 16.701414) and Keszü (46.031793, 18.191574) were selected. Although the other Hungarian locations, Bogád (46.092998, 18.321933), Tiszavasvári (47.976401, 21.456783), and Szeged (46.306404, 20.121326), were not individually featured, the high-definition satellite images at the coordinates demonstrated in a clearly visible way the existence and character of shading by the examined objects in the entire territory of Hungary. In Táplánszentkereszt, a satellite image of such excellent quality was captured of the surface on 2 January 2020 at approximately 11:30 a.m. (local time), in which the shading effects on the area were extremely clearly visible. At the site, Fenyő-type transmission towers can be seen, and the conductors belonging to each cross-arm can be seen well in the case of a Sun height of approximately 20° [81], casting a slightly arched shadow on the surface. The shadow of the tower was about 126 m long (Figure 3). These results confirm that, in the planning of PV power plants, it is not only shading by the transmission towers that needs considering but also shading by the conductors, so that the most suitable PV power plant construction strategies can be selected. It may also occur in the case of PV power plant investments that the size and the unique characteristics of a given site require taking more risks. In the case of Keszü, an 11 MWp PV system was built with conventional PV modules at a location traversed by Fenyő transmission towers in 2020. Figure 4 demonstrates the extent of the shading of the strings that a layout which is riskier from the perspective of PV module lifetime can cause. In addition, it also confirms the assumption that, in the course of designing a PV power plant, it is of utmost importance to explore the specific conditions of the potential construction sites thoroughly, since this is how the risk factors connected to the building of the PV power plant can be taken into account and their effects decreased.

**Figure 5:**
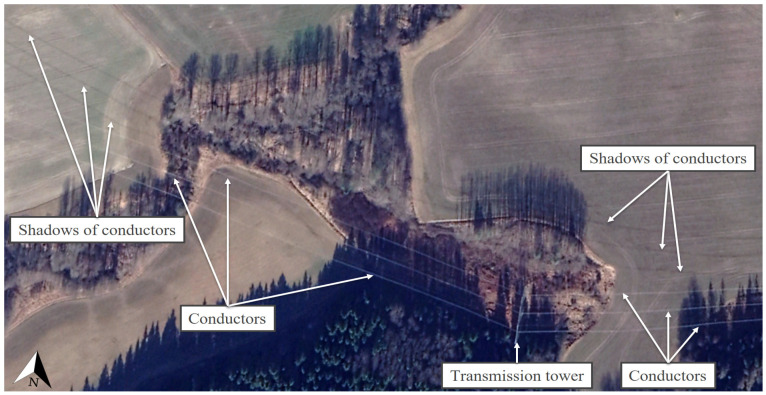
The shading characteristics of high-voltage transmission tower and its conductors in Botkyrka, Sweden, on 19 March 2020 at approximately 1:00 p.m. (local time), based on [41].

**Figure 6:**
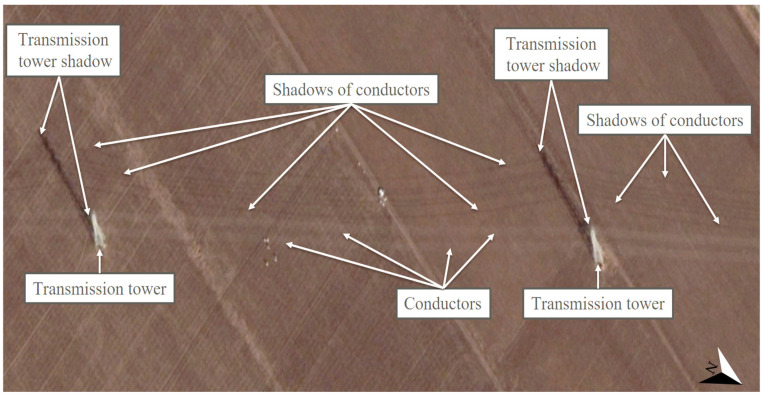
The shading characteristics of high-voltage transmission tower and its conductors in Buin, Chile, on 10 July 2019 at approximately 8:00 a.m. (local time), based on [41].

It is important to note that the shading effects demonstrated in Figure 3 and Figure 4 can also be observed globally. High-voltage transmission towers and their conductors can cause similar shading issues not only in the northern (Sweden, Botkyrka S, Figure 5) but also in the southern (Chile, Santiago, Figure 6) hemisphere. The satellite images in Figure 3, Figure 4, Figure 5 and Figure 6 can attest that it is not only shading by transmission towers that needs to be considered but also that of their conductors when designing PV power plants, since the shadows of the conductors belonging to each cross-arm could be clearly seen between the high-voltage transmission towers in winter, when the Sun was low. This is important information because, on the one hand, it was never previously proven that conductors cast clearly visible shadows on the surface, and, on the other hand, in Hungary, the time from 9:00 a.m. to 2:00 p.m. (UTC + 1) at the winter solstice is the standard for determining the shading characteristics of objects problematic from the point of view of project sites [37].

### 3.3. The Validation Results of the Simulation Procedure Used in the Modeling

**Figure 7:**
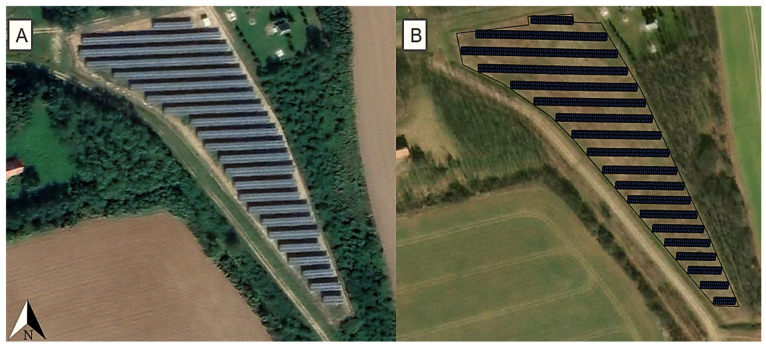
The layouts of the actual (**A**) and the modelled (**B**) PV power plants with equal capacities of 0.5 MW in Kiskölked, Rádóckölked, based on [41,54,55].

As modeling requires the validation of the simulation procedures used, the validation of the procedures herein was carried out with the help of the Skelion and SketchUp Pro programs at the site of a PV power plant of a nominal power of 0.5 MW (Kiskölked, Rádóckölked, 47.069888, 16.580578). The capacity of the PV power plant created by the modeling method was 0.5 MW, which was equal to the 0.5 MW power of the actual PV power plant. Thus, it was proven that the methodology used was suitable for the reliable surveying of the potential sizes of PV power plants at individual locations (Figure 7). The inter-row spacing of the PV power plant in the model differed somewhat from the actual spacing, because the program used distances adjusted to the relief of the area.

### 3.4. The Identification of the Unshaded and Shaded Zones Belonging to Each Scenario of the Examined Area

**Figure 8:**
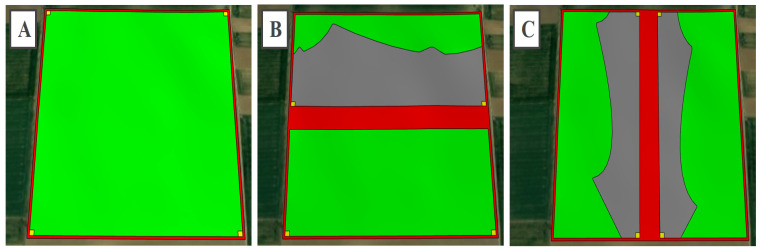
The unshaded and shaded zones, the 40 m^2^ safety zones, and the compulsory distances belonging to each scenario of the examined area marked in different colors: (**A**) area of scenario A; (**B**) area of scenario B; (**C**) area of scenario C. Color codes: red, area loss due to area needs of fences, high-voltage transmission towers, their conductors and bases, and compulsory distance; gray, shaded zone; green, unshaded zone; yellow, 40 m^2^ safety zones for electrical substations and inverters, based on [54,55].

The shading situation seen at the site in Táplánszentkereszt showed that each conductor belonging to the arms of the 46 m tall transmission tower cast a slightly arched shadow on the surface when the Sun was at a height of 20° (Figure 3). It was established by using SketchUp Pro that the lowest point of the conductors was at 65% of the height of the transmission tower; thus, the model took this ratio and the arched character according to this into account. The unshaded and shaded zones, the 40 m^2^ safety zones, and the compulsory distances belonging to each scenario of the 22.7 ha area (Juta, 46.403736, 17.719469) can be seen marked in different colors in Figure 8. This figure demonstrates clearly that high-voltage transmission towers and their conductors pose a shading risk even in the case of a larger area, also emphasizing the importance of exploring the unique features of a potential investment site. It is visible that the shadows cast by the transmission towers and their conductors were of an irregular shape, which further justifies the necessity of the exploration of the advantages and disadvantages of the area, in order to create the most suitable PV power plant construction strategies. Table 5 shows the proportions of the useful area of the model site by examined scenario according to the possible mounting solutions of the conventional and the MX PV modules. The results of these made the technical and economic modeling of the different scenarios possible.

### 3.5. The Size of the PV Power Plants That Can Be Built According to the Examined Scenarios and Their Energy Production in the First Year

Related to the individual scenarios, several characteristics connected to PV power plant construction were evaluated using the Skelion program. They were the following:

- The number of the PV modules that can be placed in the unshaded and shaded zones according to the given scenario;
- The nominal power of the PV modules that can be placed in the unshaded and shaded zones according to the given scenario;
- The annual shading losses of the PV modules;
- The amount of electric energy that can be sold yearly from the unshaded and shaded zones in the case of a 1 kWp PV system;
- The amount of electric energy produced by the PV modules mounted in the unshaded zones, the shaded zones, and the whole site that can be sold in the first year.

The evaluated characteristics (Table 6) show the advantages and disadvantages regarding the PV module arrangement solutions of the individual scenarios in detail.

Scenario A is the most beneficial version from the perspective of the PV power plant size (16.6 MWp) and the resultant annual electric energy production (20.1 GWh), since the site is not affected by shading.

In the case when only the unshaded area is utilized with the help of conventional PV modules (scenarios B2, C2), the east–west shading direction allows the construction of a larger PV power plant (scenarios B2: 10.1 MWp, C2: 9.7 MWp). Contrary to this, when both the unshaded and the shaded zones are utilized (scenarios B1, B3, C1, and C3), the northern shading direction offers the greater potential for constructing PV power plants (B1: 14.8 MWp, B3: 14.9 MWp, C1: 15.3, C3: 15.4). In addition, in the case of northern shading, the annual shading loss of the traditional and MX PV modules placed in the shaded zone is less.

In the case of scenarios C1 and C3, it can be observed that the spacing distance between the strings in the shaded zone is more favorable than in scenarios B1 and B3. This is because dividing the area in a north–south direction resulted in shorter rows, fitting the character of the relief better, as well as a spacing distance more optimal for the utilization of the site (Table 6). Correspondingly, in scenarios C1 and C3, the PV power plants that can be built are of 15.3 MWp and 15.4 MWp, respectively, while, in the cases of B1 and B3, they are of 14.8 MWp and 14.9 MWp, respectively.

### 3.6. The Economic Aspects of the Examined PV System Scenarios

Investment profitability analyses were performed for each scenario. At the bottom of Table 6, the amount of electric energy that can be sold in the first year from the examined PV power plants (GWh) is shown. This information provides the basis of the 15 year economic analysis. Table 7 presents the economic indicators calculated for every case in detail. It can be seen in Table 7 that every investment was proven to be profitable during the examined period. It is apparent that high-voltage transmission towers and their conductors, which are problematic from the perspective of shading, can significantly influence the construction strategy of a PV power plant and the profitability characteristics of the investment related to it.

The ideal case is when there are no shading problems at the site (scenario A), since this situation allows the minimization of the annual performance degradation of PV the modules, as well as the maximization of the number of the conventional PV modules and the amount of energy that can be sold from the whole area.

In the case when the traditional PV modules were mounted only in the unshaded zone and the rest remained unutilized (scenarios B2 and C2), the IRR and the DPP were the same as in scenario A, but the amount of PV modules that could be installed was significantly reduced. This is disadvantageous because most of the area (scenario B2: 28.4%, scenario C2: 31.3%) remains unused and, thus, the quantity of the electric energy that can be generated is also greatly decreased compared to scenario A.

Shading in an east–west direction (scenarios B1 and B3) had a more positive effect on the investment profitability indicators (due to rounding, this is only visible in the IRR in Table 7). However, it affected the size of the PV power plant in a more negative way compared to the northern one (scenarios C1 and C3). This was because dividing the area in a north–south direction resulted in more optimal distances between the rows.

**Figure 9:**
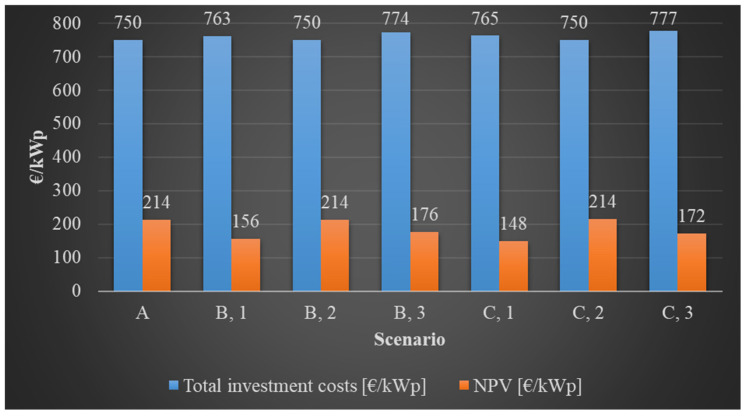
Comparison of the different investment alternatives studied and converted to a 1 kWp system.

**Figure 10:**
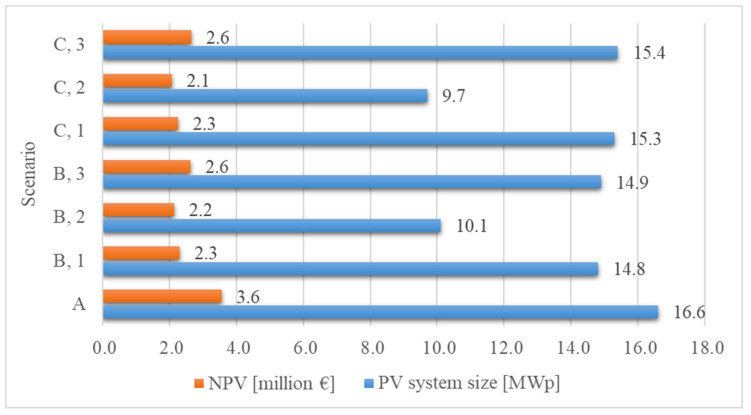
PV system sizes and the NPV figures related to them by scenario.

The investigations confirmed that, in the case of the utilization of the shaded areas, using the MX solution allowed not only the building of a larger PV power plant compared to the conventional PV module technology, but the profitability indicators of the investment (NPV and IRR) proved to be more favorable too. This means that, in the event of shading issues, the use of MX modules was economically more favorable in contrast to traditional technologies. The primary reason for this is the faster speed of degradation of conventional technologies caused by shading and the resultant need for PV module replacement. Figure 9 illustrates the profitability results of the investment, and it shows the NPV figures for the total investment cost per 1 kWp by scenario. It can also be seen here that, in the event of shading problems, it is more reasonable to use the MX solution. Illustrating the total PV power plant sizes and the NPVs related to them by scenario provides important information regarding the investment (Figure 10). The combined presentation of these makes it possible to compare the NPV for the whole area with the PV power plant size, i.e., the nominal power that can be built potentially, for each scenario. The latter information is also important for the investor if they do not want to assess the investment from a solely financial perspective and the quantity of generated electric energy is also of significance (e.g., because of future expectations). It is apparent that, because of the shading effects of the transmission towers and their conductors, the exploration of the advantages and disadvantages of the area is justified in order to create the most suitable PV power plant construction strategies.

## 4. Discussion

Currently, the monocrystalline technology is the most popular in terms of conventional PV modules for new PV power plant investments, since its module cost in EUR/Wp is practically equal to that of the polycrystalline technology. In addition, sites selected for investments can be used more efficiently when using monocrystalline PV modules because of their higher efficiency. The disadvantage of conventional crystalline PV technologies is that their module cells are sensitive to unfavorable effects that cause differences in performance among the PV cells. Such an effect may be, for example, partial shading, when highly localized dissipation occurs inside the PV modules because of electric power turning into heat. However, at present, there is already a technological solution that can efficiently handle this phenomenon and the problems of loss caused by shading. It is an increasing demand by investors that the sites selected for PV power plant investments be surveyed from every aspect in order to identify all the risk factors and the suitable technological solutions. From the perspective of shading, high-voltage transmission towers and their conductors can greatly influence the profitability characteristics of a PV power plant investment, as these objects have extremely great and significant shading effects. This is why the thorough investigation of the effects of these objects on PV power plants and the exploration of the relationships has now become inevitable. This study has proven that, because of the shading effects of the transmission towers and their conductors, the exploration of the advantages and disadvantages of a given area is justified in order to develop the most suitable PV power plant construction strategy.

The original goal of the study was to create a generally valid model. However, it was found that the building sites and economic aspects of every PV power plant project were all unique. Consequently, it is impossible to create a model of general validity, but the research explored those technical and economic approaches and applied software solutions that can provide help with the formulation of the technical and economic aspects of suitable PV power plant construction strategies in Hungary.

## 5. Conclusions

In the design of photovoltaic (PV) systems, it is important to be aware of the country-specific economic, environmental, market, political, social, and technical factors, since such investments involve considerable expenses and are meant for the long term, even several decades, due to the nature of investments in the energy sector. For the vast majority of PV power plants, investors choose the traditional crystalline PV technology, for which, however, partial shading means a significant threat, mainly because of the risk of irreversible damage to the PV modules. In the case of ground-mounted PV power plants, a great dilemma facing investors concerning the ratio of the useful area is posed by the need to take the transmission towers and their conductors into consideration, since their shading effects are extremely great and significant.

With regard to Hungary, this study explored, in connection with the problems of shading by high-voltage transmission lines, the risk factors important for PV power plant investments, as well as their technical and economic aspects and the possible solutions. Concerning Hungary, there are no research results available currently that are related to the investigations presented in this paper. Although some international research has touched upon certain aspects of the matter (e.g., Dolara et al., 2016 [29]; International Energy Agency, 2018 [17]; Chen et al., 2020 [14]), its complexity was not scrutinized. In addition, the results of the investigations herein can provide help with the formulation of the most suitable PV power plant construction strategies in Hungary and all around the world.

The satellite images shown in the manuscript provide proof that phenomena similar to the shading effects caused by high-voltage transmission towers and their conductors in Hungary can be seen at any point of the Earth in the winter season at low Sun elevations. Thus, it can be stated that the results obtained regarding the issue of shading related to high-voltage transmission towers can also be deemed generally valid internationally. It was verified that the shadows cast by the transmission towers and their conductors are of an irregular shape, which further justifies the necessity of the exploration of the advantages and disadvantages of the area, in order to create the most suitable PV power plant construction strategies possible. The most important globally valid (i.e., applicable not only to Hungary) finding of the study is that not only the shading effects of high-voltage transmission towers but also those of the conductors between them need to be taken into account in order to prevent the hot spot effect and to utilize the area in an optimal way.

## Figures and Tables

**Table 1:** The description of the main aspects of the modeling.

Description	Values							Ref.
Tilt angle of PV modules (°)	30							[7,10]
Orientation (azimuth) (°)	180							[10,53]
Layout of the PV modules	3 rows, horizontal							[10]
Decrease in annual performance of traditional crystalline modules after the first year, without shading (%)	0.5							[58,59]
Decrease in annual performance of traditional/MX crystalline modules after the first year, in the case of shading (%)	2.5/0.8							[20,32,60]
Area of analyzed site (ha)	22.7							-
Distance from fence (m)	5							[10]
Height of fence (m)	2							[37]
Distance from outer conductors of high-voltage line on both sides (m)	18–18							[10,61]
Distance between high-voltage transmission towers distance (m)	325							[41]
Reference date and time (local time) for PV string spacing	21 December 2020, 11:47 a.m.							[53]
Reference date and time (local time) for determining shading zones of high-voltage transmission towers and conductors	21 December 2020, 9:00 a.m.–2:00 p.m.							[37]
Examined scenario	A	B1	B2	B3	C1	C2	C3	-
Do high-voltage transmission towers cross the middle of the site?	no	yes, only in an east–west direction			yes, only in a north–south direction			-
Can shading be observed on the PV modules during the year?	no	yes, only in shaded area	no	yes, only in shaded area	yes, only in shaded area	no	yes, only in shaded area	-
Installing conventional PV modules?	yes, in whole area	yes, in whole area	only in unshaded area	only in unshaded area	yes, in whole area	only inunshadedarea	only in unshaded area	-
Installing MX PV modules?	no installation	no installation	no installation	only in shaded area	no installation	no installation	only in shaded area	-
PV module failure and resultant replacement?	no	yes, every 3 years, 3% of PV modules in shaded zone	no	no	yes, every 3 years, 3% of PV modules in shaded zone	no	no	-

**Table 2:** The main context of the economic calculations of the modeling.

Description	Context	Ref.
Annual PV system energy output (kWh/a)	First year, all scenarios: $E_{1}=$ the software results of Skelion	Based on [66]
	Other years, traditional PV modules without shading effects: $E_{t}=E_{\left(t-1\right)}\left(1-0.005\right)$; Other years, traditional PV modules with shading effects: $E_{t}=E_{\left(t-1\right)}\left(1-0.025\right)$ Other years, MX PV modules with shading effects: $E_{t}=E_{\left(t-1\right)}\left(1-0.008\right)$	
Total cash flow (EUR)	$CF_{total}=\left[\left(E_{total}\;Selling\;price{\;}_{total})+\;(Sales\;of\;PV\;modules,\;inverters\;and\;mounting\;systems\right)\right]-(C_{0}$+$\;C_{O\&Mtotal}+Scheduling\;fee+Disassembling\;cost)$	
Net present value (EUR)	$NPV=-C_{0}+\overset{n}{\underset{t=1}{\sum }}\frac{C_{t}}{{\left(1+r\right)}^{t}}$	Based on [66,76,77]
Internal rate of return (%)	$0=-C_{0}+\overset{n}{\underset{t=1}{\sum }}\frac{C_{t}}{{\left(1+IRR\right)}^{t}}$	
Discounted payback period (EUR)	$C_{0}+\overset{DPP}{\underset{t=1}{\sum }}C_{t}=0$	

**Table 3:** Economic figures used in the calculations.

Description	Values	Ref.
Instalment and realization of PV power plant without PV modules (EUR/kWp)	420	[10,78]
Jinko Cheetah JKMS315M-60B PV module price (EUR/pcs)	104	[35]
JKMS320M-60HB-MX3 PV module price (EUR/pcs)	129	[36]
Selling price of electric energy in the first year (EUR/kWh)	0.0595	[49]
Average rate of inflation (2015–2019) (%)	2.29	[65]
Bond yield (%)	2.26	[67]
Price of 1 kWp of used crystalline PV module, fourth quarter of 2020 (EUR)	112.5	[68]
Selling price of mounting systems (EUR)	25% of the selling price of used PV modules	[70]
Price of 1 kWp of used central PV inverter, fourth quarter of 2020 (EUR)	20	[69]
Financial support (%)	0	-
Scheduling costs in the first year in the case of 1 MWp system (EUR)	2640	[37]
Increase in scheduling costs until 2026 (%)	10	[75]
Operating and maintenance tasks of PV power plant (EUR/MWp)	3000	[79]
Costs of disassembling PV power plant at the end of the 15th year	8% of the costs of installation and realization of PV power plant	[79]

**Table 4:** The height characteristics of Hungarian high-voltage transmission towers.

Description	Values
The minimum height of Hungarian high-voltage transmission towers (m)	20.5
The maximum height of Hungarian high-voltage transmission towers (m)	53.2
The median height of Hungarian high-voltage transmission towers (m)	35.4
The average height of Hungarian high-voltage transmission towers (m)	36.6
The standard deviation of the heights of Hungarian high-voltage transmission towers (m)	7.8
The coefficient of variation of the heights of Hungarian high-voltage transmission towers (CV) (%)	21.4

**Table 5:** The proportions of the useful area of the model site by examined scenario according to the given mounting solutions of the conventional and MX PV modules.

Description	Values						
Examined scenario	A	B1	B2	B3	C1	C2	C3
Percentage of useful area with conventional PV modules in unshaded zone (%)	95.7	57.3	57.3	57.3	54.2	54.2	54.2
Percentage of useful area with conventional PV modules in shaded zone (%)	-	28.4	-	-	31.3	-	-
Percentage of useful area with MX PV modules in the shaded zone (%)	-	-	-	28.4	-	-	31.3
Percentage of total useful area with PV modules (%)	95.7	85.7	57.3	85.7	85.5	54.2	85.5
Percentage of area loss (%)	4.3	14.3	42.7	14.3	14.5	45.8	14.5

**Table 6:** Features of the PV power plants of the examined scenarios by the possible construction solutions of the conventional and MX PV modules.

Description	Values						
Examined scenario	A	B1	B2	B3	C1	C2	C3
Number of conventional PV modules that can be placed in unshaded zone (pcs)	52,761	31,950			30,639		
Number of conventional PV modules that can be placed in shaded zone (pcs)	-	15,063	-	-	17,838	-	-
Number of MX PV modules that can be placed in shaded zone (pcs)	-	-	-	15,063	-	-	17,838
Nominal power of conventional PV modules that can be placed in unshaded zone (MW)	16.6	10.1			9.7		
Nominal power of conventional PV modules that can be placed in shaded zone (MW)	-	4.7	-	-	5.6	-	-
Nominal power of MX PV modules that can be placed in shaded zone (MW)	-	-	-	4.8	-	-	5.7
**Nominal power of PV power plant that can be built in the whole area (MW)**	**16.6**	**14.8**	**10.1**	**14.9**	**15.3**	**9.7**	**15.4**
Total annual shading loss of conventional PV modules that can be placed in unshaded zone (%)	0.05						
Total annual shading loss of conventional PV modules that can be placed in shaded zone (%)	-	4.21			3.76		
Total annual shading loss of MX PV modules that can be placed in shaded zone (%)	-	2.33			1.82		
The amount of electric energy that can be sold in the first year specifically from conventional PV modules that can be placed in the unshaded zone in the case of a 1 kWp PV system (kWh/kWp)	1211.6						
The amount of electric energy that can be sold in the first year specifically from conventional PV modules that can be placed in the shaded zone in the case of a 1 kWp PV system (kWh/kWp)	-	1161.5	-	-	1166.94	-	-
The amount of electric energy that can be sold in the first year specifically from MX PV modules that can be placed in the shaded zone in the case of a 1 kWp PV system (kWh/kWp)	-	-	-	1184.7	-	-	1190.3
The amount of electric energy that can be sold in the first year from conventional PV modules that can be placed in the unshaded zone (GWh)	20.1	12.2			11.7		
The amount of electric energy that can be sold in the first year from conventional PV modules that can be placed in the shaded zone (GWh)	-	5.5	-	-	6.6	-	-
The amount of electric energy that can be sold in the first year from MX PV modules that can be placed in the shaded zone (GWh)	-	-	-	5.7	-	-	6.8
**The amount of electric energy that can be sold in the first year from the PV power plant that can be built in the whole area (GWh)**	**20.1**	**17.7**	**12.2**	**17.9**	**18.3**	**11.7**	**18.5**

**Table 7:** The overall investment efficiency indices for the examined scenarios.

Description	Values						
Examined scenario	A	B1	B2	B3	C1	C2	C3
Studied investment period (years)	15						
PV system size (MW)	16.6	14.8	10.1	14.9	15.3	9.7	15.4
Total investment costs, net, fourth quarter of 2020 (million EUR)	12.5	11.3	7.6	11.5	11.7	7.2	11.9
Negative cash flow (CO&M,total + scheduling fee + disassembling cost, net (million EUR)	3.3	2.9	2.0	2.9	3.0	1.9	3.1
Positive cash flow, net (million EUR)	22.7	19.4	13.8	20.1	19.9	13.2	20.8
NPV (million EUR)	3.6	2.3	2.2	2.6	2.3	2.1	2.6
IRR (%)	5.46	4.62	5.46	4.86	4.51	5.46	4.79
DPP (year)	13	14	13	14	15	13	14

## Data Availability

The data presented in this study are available within the article.

## Abbreviations

The following abbreviations are used in this manuscript:

C_0_	Total initial investment costs (EUR)
CF_total_	Total cash flow (not discounted) (EUR)
C_O&M,total_	Total operation and maintenance costs for the duration of the investment (EUR)
C_t_	Discounted (net) cash inflow for the duration of investment (EUR)
CV	Relative standard deviation (%)
DC	Direct current (A)
DPP	Discounted payback period (years)
E	Annual PV energy output (kWh)
E_1_	Annual PV energy output in first year (kWh)
E_t_	Annual PV energy output in other years (kWh)
E_total_	Total PV energy output for the duration of the investment (kWh)
IRR	Internal rate of return (%)
NPV	Net present value (EUR)
IC	Integrated circuit
KÁT	Hungarian system of supporting green energy from renewable energy sources until 31 December 2016
MAVIR	Hungarian Transmission System Operator
MEKH	Hungarian Energy and Public Utility Regulatory Authority
METÁR	Renewable Energy Support Scheme from 1 January 2017
MX	Maxim Integrated
PV	Photovoltaic
